# Identification of Antigenic Proteins of the Nosocomial Pathogen *Klebsiella pneumoniae*

**DOI:** 10.1371/journal.pone.0110703

**Published:** 2014-10-21

**Authors:** Sebastian Hoppe, Frank F. Bier, Markus von Nickisch-Rosenegk

**Affiliations:** 1 Department of Bioanalytics and Biosensorics, Fraunhofer Institute for Cell Therapy and Immunology, Branch Bioanalytics and Bioprocesses (IZI-BB), Potsdam, Germany; 2 Institute of Biochemistry and Biology, University of Potsdam, Potsdam, Germany; 3 Department of Biosystem Integration and Automation, Fraunhofer Institute for Cell Therapy and Immunology, Branch Bioanalytics and Bioprocess (IZI-BB), Potsdam, Germany; Université d'Auvergne Clermont 1, France

## Abstract

The continuous expansion of nosocomial infections around the globe has become a precarious situation. Key challenges include mounting dissemination of multiple resistances to antibiotics, the easy transmission and the growing mortality rates of hospital-acquired bacterial diseases. Thus, new ways to rapidly detect these infections are vital. Consequently, researchers around the globe pursue innovative approaches for point-of-care devices. In many cases the specific interaction of an antigen and a corresponding antibody is pivotal. However, the knowledge about suitable antigens is lacking. The aim of this study was to identify novel antigens as specific diagnostic markers. Additionally, these proteins might be aptly used for the generation of vaccines to improve current treatment options. Hence, a cDNA-based expression library was constructed and screened via microarrays to detect novel antigens of *Klebsiella pneumoniae*, a prominent agent of nosocomial infections well-known for its extensive antibiotics resistance, especially by extended-spectrum beta-lactamases (ESBL). After screening 1536 clones, 14 previously unknown immunogenic proteins were identified. Subsequently, each protein was expressed in full-length and its immunodominant character examined by ELISA and microarray analyses. Consequently, six proteins were selected for epitope mapping and three thereof possessed linear epitopes. After specificity analysis, homology survey and 3d structural modelling, one epitope sequence GAVVALSTTFA of KPN_00363, an ion channel protein, was identified harboring specificity for *K. pneumoniae*. The remaining epitopes showed ambiguous results regarding the specificity for *K. pneumoniae*. The approach adopted herein has been successfully utilized to discover novel antigens of *Campylobacter jejuni* and *Salmonella enterica* antigens before. Now, we have transferred this knowledge to the key nosocomial agent, *K. pneumoniae*. By identifying several novel antigens and their linear epitope sites, we have paved the way for crucial future research and applications including the design of point-of-care devices, vaccine development and serological screenings for a highly relevant nosocomial pathogen.

## Introduction

*Klebsiella pneumoniae* is a gram-negative, facultative anaerobic rod-shaped bacterium belonging to the family of Enterobacteriaceae. It is a non-motile, lactose fermenting organism, which has been known to cause severe lung damage if aspirated. Other clinical symptoms common with *Klebsiella pneumoniae* infections encompass urinary-tract-infections (UTI) and wound infection potentially causing bacteremia and septicemia [1]. In recent years it has become one of the most persistent nosocomial agents, especially due to the increasing distribution of multiple resistances to antibiotics. The most prominent group of *K. pneumoniae* harboring a broad resistance spectrum incorporates the Extended-spectrum beta-lactamase (ESBL) expressing strains. Due to their outstanding clinical relevance and occurrence as agents of nosocomial infections, it is highly desirable to rapidly detect the presence of these organisms and to find suitable measures to effectively counter any infection in the early stages [2]. While numerous DNA-based typing methods exist [3], these are often laborious and time-consuming. In contrast, user-friendly point-of-care devices applying antigen-antibody interactions would allow for a quick and reliable detection [4]. Nevertheless, the knowledge about suitable antigens to be incorporated into such a device is scarce. Thus, we have utilized a method to quickly assess novel immunogenic proteins of *K. pneumoniae*, which might serve as potential targets for a diagnostic tool.

Recently, we have successfully employed this approach to unveil immunogenic proteins for both *Campylobacter jejuni* [5] and *Salmonella enterica* [6].

Concisely, prokaryotic cDNA libraries are created, fusion proteins expressed and these constructs covalently attached to microarray surfaces via the use of a HaloTag (Promega). Subsequently, the microarrays are screened using polyclonal antibodies reactive to the donor species of the cDNA. Therefore, this approach enables a broad and reliable screening, while reducing cross-reactivity and background to a minimum [7] due to the highly selective and covalent binding of HaloTag to its specific ligand [8]. Moreover, the high specificity of said interaction renders excessive protein purification steps normally encountered in microarray-based screening applications [9] obsolete. Thus, it is a faster and more direct approach as spotting combines both the deposition of samples and enables the immediate purification by a simple washing step.

In connection with the above screening approach, cDNA derived expression libraries were generated to express a vast number of proteins from *K. pneumoniae*. These libraries offer the advantage of a smaller sample size as compared to genomic libraries. This is mainly due to genomic libraries encompassing highly truncated DNA fragments as well as DNA representing regions that do not encode proteins in the original organism. Contrastingly, cDNA libraries generated via the In-fusion SMARTer Directional cDNA Library Construction Kit (Clontech) have been known to lead to longer fragments and possess a high abundance of full-length clones [10].

Thus, the overall number of clones required for screening is substantially reduced.

Still, prokaryotic cDNA libraries display one main disadvantage. As bacterial mRNA rarely contains a Poly(A)-tail [11], [12], isolation of the mRNA from other RNA species is tedious. While some methods exist to isolate the mRNA prior to reverse transcription [13], [14], we rather chose to normalize the cDNA afterwards. Consequently, the entire RNA of *K. pneumoniae* was used for reverse transcription. Next, normalization was performed using a Duplex-specific nuclease [15]. This treatment has been shown to effectively reduce the highly abundant rRNA derived cDNA portions without implementing a bias, thus altering the overall composition of the cDNA in favour of the mRNA derived molecules [16]. In addition to this, ligation-independent cloning and electroporation were employed to enhance cloning efficiency [17].

In this work, we have screened 1536 clones to detect the presence of previously unknown immunogenic proteins. In summary, we identified 14 proteins that have not been described as immunogenic before. After further analyses and epitope mapping of several promising candidates, three proteins – a channel receptor, a putative transport protein and a hypothetical protein – revealed linear antigenic sites with varying specificity.

Our results offer the potential to be used for a wide array of applications including the generation of monoclonal antibodies that might be used in diagnostic examination. Furthermore, several of the identified antigens or parts thereof might be suitable for vaccine development, either used in passive or active immunization. Additionally, many virulence-associated factors harbor some immunogenic potential. Thus, identification of novel immunogenic proteins might elucidate proteins involved in the pathogenicity and virulence of *K. pneumoniae*. Consequently, this advances the understanding of this pathogen and illuminates new approaches to counter infections.

## Results

### Screening of cDNA expression library

The mean RIN value of RNA used for cDNA generation and library construction was calculated to 7.3±1.3 (n = 5). After successful normalization, the cDNA was cloned to create an expression library. Of this library, 1536 different clones were screened using HaloLink slides. The known immunogenic proteins [18], outer membrane protein 1a OmpF (UniProt/Swiss-Prot: A6T721) and outer membrane protein 3a OmpA (A6T751), were used as positive references, whereas both dihyroorotase PyrC (A6T7D6) and glyceraldehye-3-phosphate-dehydrogenase GapA (A6T8L2) served as negative references. After screening, the signal intensities of each sample were compared to the references and grouped accordingly. Generally, three distinct groups were established. Group I represents samples exceeding the intensities of both positive reference proteins, group II encompasses samples ranging in between the different intensities of OmpA and OmpF, whereas group III entails those samples that albeit showing higher intensities than the negative controls, are below both OmpA and OmpF. Here, approximately 25% of samples belong to group I, while II and III contain 7% and 14% respectively. The remaining samples, 54%, fall into the same range as the negative protein references. Consequently, 192 clones or 12.5% of the entire screening approach were selected for sequencing. These clones were all taken from group I. After sequencing, artificial fragments and known antigens were discarded to reveal potentially novel immunogenic proteins, see Table S1 for a list of the initial identification via sequencing. Some of the inserts were too heavily truncated to reliably identify the corresponding gene. Moreover, in some cases subcloning the initially identified genes in full-length failed even after numerous attempts. Therefore, those genes were removed from further characterization as the translated peptide fragments were too short to be significant. Despite these limitations, 14 potentially novel immunogenic proteins were expressed in full length and used for further characterization.

### Characterization and analysis of immunodominant behaviour

The 14 antigen candidates are summarized in Table 1 including their locus tag, protein name, length and size in kDa. After recombinant expression, the presence of each fusion construct was analyzed via SDS-PAGE employing a fluorescently-labelled HaloTag Alexa Fluor 488 ligand, see Figures S1 and S2. Fusion constructs harboring the correct size, i.e. 34 kDa added to the values listed in table 1 due to the attached HaloTag, are highlighted by red boxes. For KPN_00459, a putative transport protein, and KPN_00466, a hypothetical protein, bands of the correct size are hardly visible indicating a low expression, if at all. In contrast, the remaining samples revealed bands of various intensity of the correct size indicating diverse expression levels. Moreover, bands at roughly 34 kDa in size are visible throughout, likely indicative of lone HaloTag without the attached fusion partner.

**Table 1:** Immunodominant protein candidates of *K. pneumoniae*.

Locus Tag [UniProt/Swiss-Prot]	Protein	length [aa]	Size [kDa]	Mean Q ± s.d. [n = 10]
KPN_00182 [A6T4X1]	30S ribosomal protein S2	241	26.7	0.66±1.22
KPN_00363 [A6T5E4]	Nucleoside channel; receptor of phage T6 and colicin K	294	33.5	0.77±0.14
KPN_00459 [A6T5N9]	Putative transport protein	558	59.3	0.83±0.27
KPN_00466 [A6T5P6]	Hypothetical protein	152	16.6	1.15±0.22
KPN_00786 [A6T6K1]	Putative AcrB Cation/multidrug efflux pump	1021	111.5	0.47±0.13
KPN_01100 [A6T7G2]	Histidine triad (HIT) protein	118	13.1	1.31±0.27
KPN_01584 [A6T8U4]	Hypothetical protein	113	12.9	1.90±0.36
KPN_01784 [A6T9E4]	Phosphoglycerate mutase	206	22.8	0.96±0.21
KPN_02199 [A6TAK6]	CoA-linked acetaldehyde dehydrogenase and iron-dependent alcohol dehydrogenase; pyruvate-formate-lyase deactivase	891	95.8	2.16±0.39
KPN_02202 [A6TAK9]	Glucose-1-phosphate uridylyltransferase	300	32.6	1.69±0.56
KPN_02919 [A6TCK4]	Putative yhbH sigma 54 modulator	109	12.3	1.39±0.38
KPN_03356 [A6TDT2]	D-erythrose-4-phosphate dehydrogenase	342	37.4	1.49±0.50
KPN_03668 [A6TES5]	50S ribosomal protein L11 methyltransferase	293	31.8	1.70±0.38
KPN_03732 [A6TEY5]	Peptidyl-prolyl cis-trans isomerase	276	29.4	1.73±0.66

The table lists 14 novel immunodominant proteins. The mean Q value (n = 10) was calculated and represents the respective signal intensity normalized to the median intensities of known antigens, OmpA and OmpF. The associated error was determined using error propagation by Gauss.

The difference in immunodominant behaviour was assessed by ten independent microarray and ELISA analyses employing two different antibodies. In summary, Table 1 reveals the resulting mean Q values and corresponding errors (n = 10). A Q value above one represents higher intensity signals than OmpA, the used positive reference. The highest mean Q value was obtained for KPN_02199, a CoA-linked acetaldehyde dehydrogenase and iron-dependent alcohol dehydrogenase; pyruvate-formate-lyase deactivase with 2.16±0.39. However, as this protein is highly conserved within all bacteria, it was not considered for further analyses regarding specific antigenic sites. The same holds true for KPN_03668, the 50S ribosomal protein L11 methyltransferase. Although a Q value of 1.70±0.38 was attained, the highly conserved nature of this protein renders it unsuitable within a diagnostic approach. Contrastingly, KPN_00466 and KPN_01584, two hypothetical proteins, were selected for future investigations as little is known about the function of these proteins. Their mean Q values were 1.15±0.22 and 1.90±0.36 respectively. Moreover, KPN_01584 shows some homology to a known superantigen of *Yersinia pseudotuberculosis* according to the NCBI Protein Cluster database [19]. Thus, the potential utilization of these proteins within a diagnostic tool seems plausible. In addition, KPN_01100, a histidine triad protein, KPN_02202, Glucose-1-phosphate uridylyltransferase, KPN_00363, a nucleoside channel and receptor of phage T6 and colicin K and KPN_00459, a putative transport protein, were selected for further investigations via epitope mapping. While KPN_01100 and KPN_02202 revealed mean Q values above one with 1.31±0.27 and 1.69±0.56, respectively, KPN_00459 and KPN_00363 failed to reach this level. Rather, the Q values attained were 0.83±0.27 and 0.77±0.14. While the Q values of the latter two proteins are lower compared to some of the other proteins identified, their functional descriptions and membrane-association render them highly attractive within a diagnostic question. Hence, they might be more easily accessible in a whole cell detection approach than cytoplasmic proteins.

### Epitope Mapping

Epitope mapping revealed the potential presence of linear epitopes within three of the six proteins investigated, namely KPN_00363, KPN_00459 and KPN_00466.

**Figure 1:**
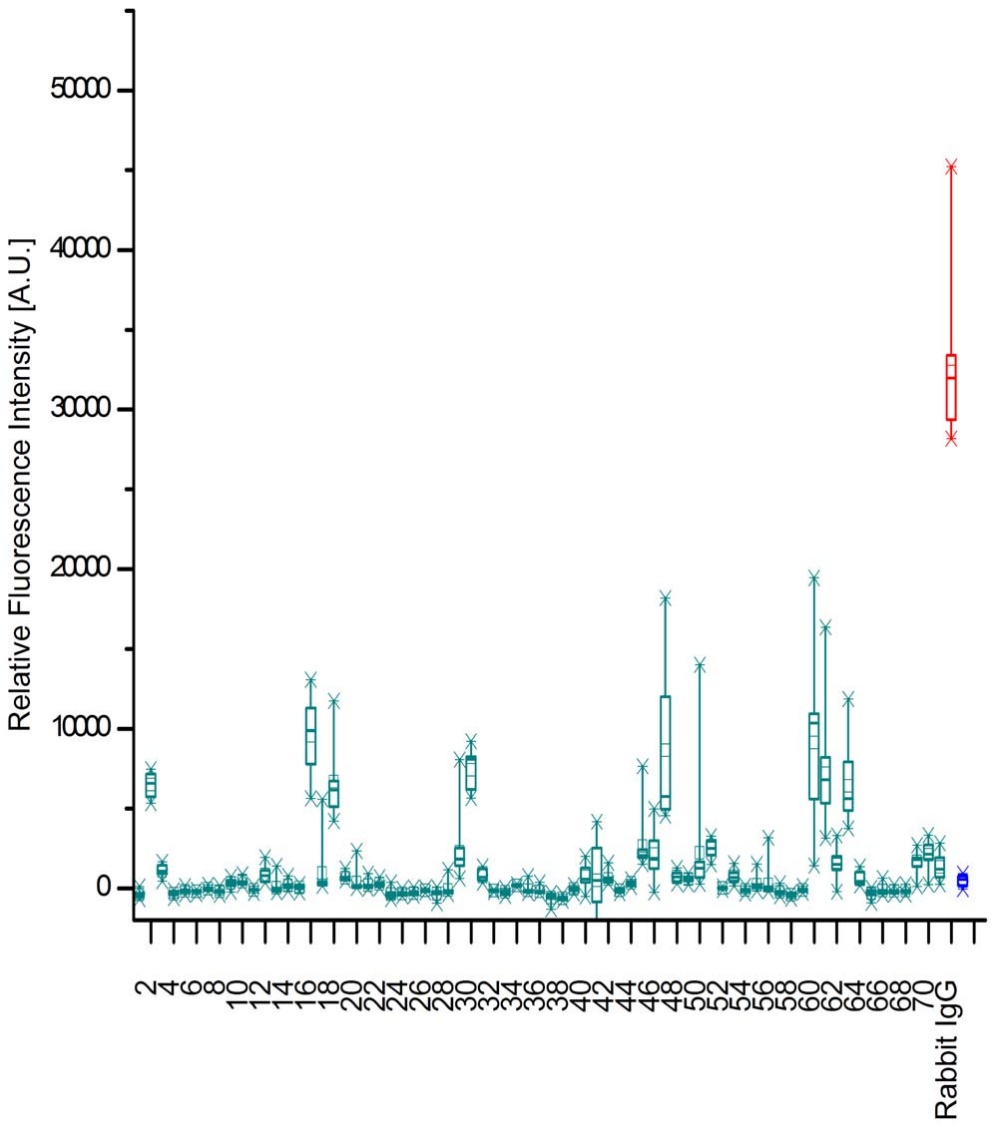
Epitope Mapping of KPN_00363. The results of the epitope mapping summarized as a box-whisker plot (n = 10). The relative fluorescence intensities of each peptide from KPN_00363 are displayed. As controls, a positive (Rabbit IgG in red) and negative reference (MBP in blue) are added to the diagram. The boxes encompass 50% of the data, while the whiskers span 98% of the values. The median is portrayed as a horizontal line and the mean embodied by a small rectangle. Seven distinct regions display intensities exceeding 1000 A.U. These include peptides 2 and 3, 16–18, 29 and 30, 45–47, 50 and 51, 60–63, and 69–71. The highest mean value of these peptides is obtained by peptide 16 amounting to roughly 10000 A.U. In contrast, the negative control MBP reached less than 500 A.U., while the positive control peaked at a mean value of more than 30000 A.U.

**Figure 2:**
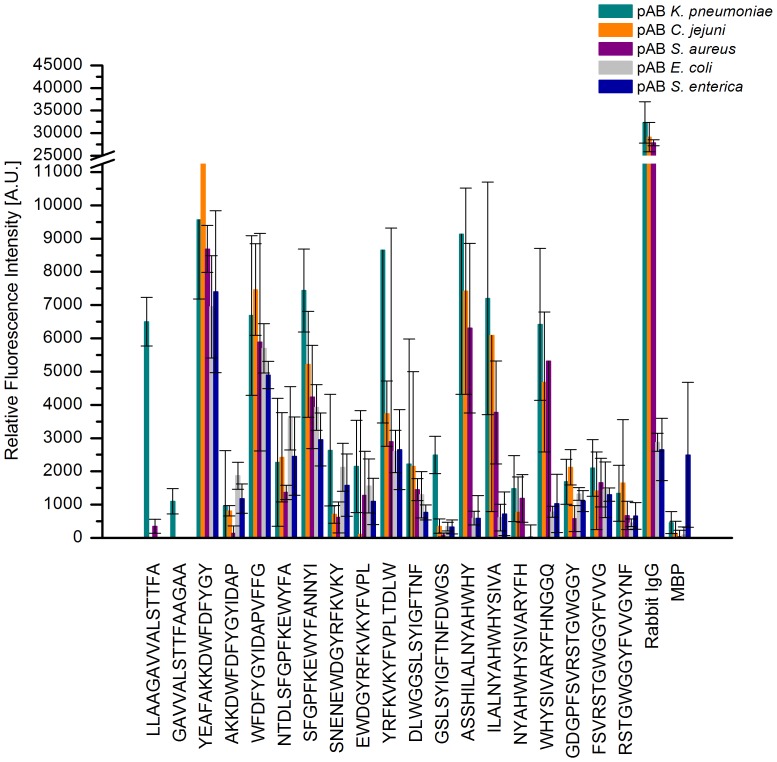
Specificity binding analysis of epitope peptides of KPN_00363. The bar chart represents the mean values [n = 6] of each potential linear epitope after incubation with polyclonal antibodies reactive to *K. pneumoniae* (green), *C. jejuni* (orange), *S. aureus* (purple), *E. coli* (grey), and *S. enterica* (blue). For peptides 2 and 3 the signal intensities after incubation with *K. pneumoniae* antibodies significantly surpassed those of the other antibody incubations. Contrastingly, all other peptides tested failed to show substantial discrimination between the different antibodies used. Mind the interruption of the ordinate for better scale.

For KPN_00363 seven distinct regions were identified with intensities above 1000 A.U., see Figure 1. These comprised the peptides 2 and 3, 16–18, 29–30, 45–47, 50–51, 60–63 and 69–71. The highest mean value for these peptides was obtained for peptide 16 with more than 10000 A.U. The positive reference, i.e. Rabbit IgG, reached a mean value of more than 30000 A.U., whereas the negative control MBP showed intensities of less than 500 A.U. As the adjacent peptides are identical in all but 4 amino acids in sequence, a consensus can easily be derived from two or more neighboring peptides. As Figure 2 reveals, only the first two peptides, LLAAGAVVALSTTFA and GAVVALSTTFAAGAA showed some specificity during specificity control assays. Here, the arrays were incubated with additional antibodies reactive to different bacterial species, namely *Campylobacter jejuni*, *Staphylococcus aureus, E. coli* and *S. enterica*. All remaining peptides display similar intensities when these antibodies were used as compared to the original *K. pneumoniae* antibodies. However, for the first two peptides a significant difference is observed. The mean value for peptide 2 was approximately 6500 A.U. with *K. pneumoniae* antibodies and dropped to less than 400 A.U. with the other antibodies. A similar trend is discernible for peptide 3, where a drop from 1100 A.U. to less than zero is visible. Thus, the consensus sequence GAVVALSTTFA is likely a suitable linear epitope featuring specificity for *K. pneumoniae*.

**Figure 3:**
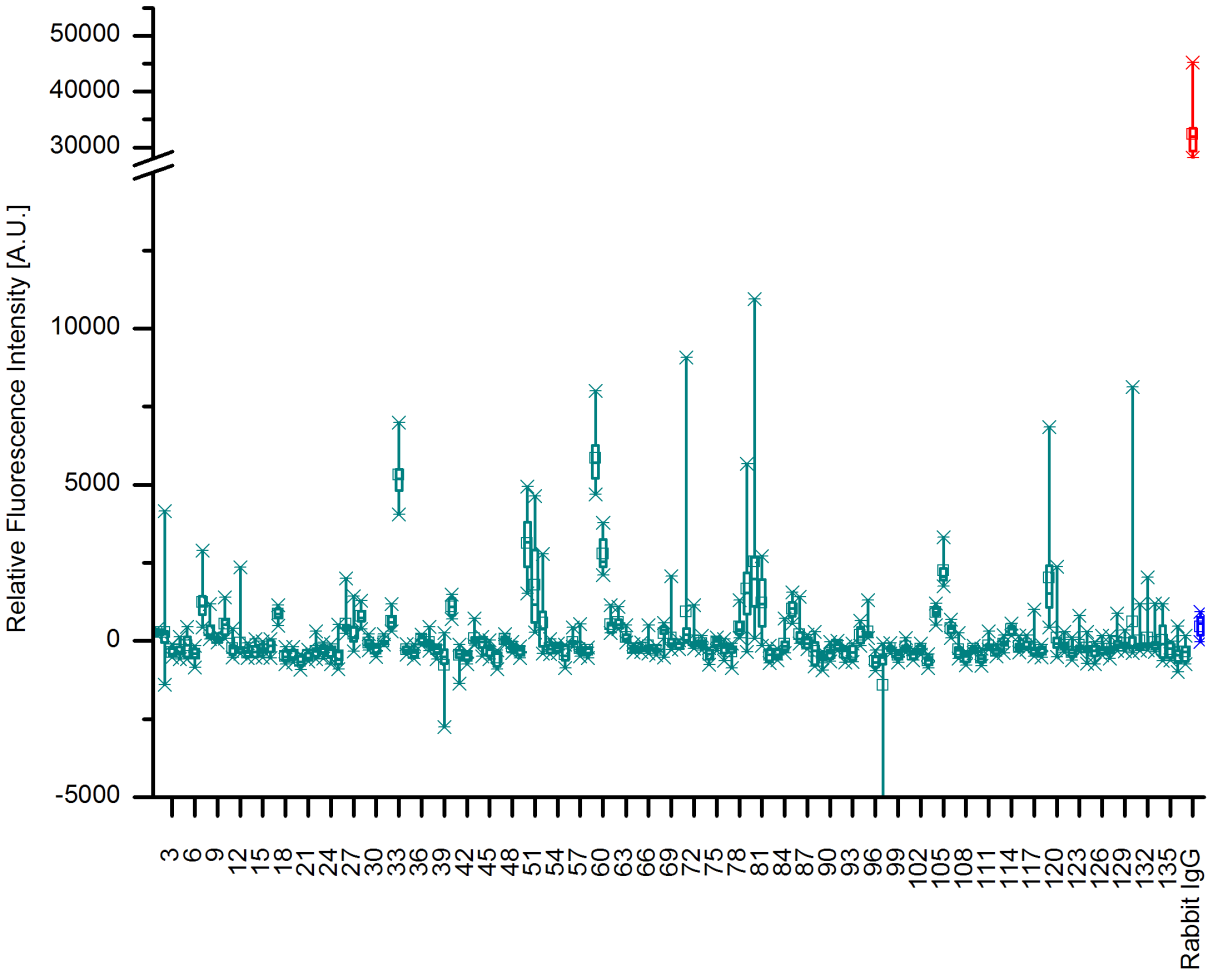
Epitope Mapping of KPN_00459. The box-whisker plot (n = 10) shows the relative fluorescence intensities of the different peptides from KPN_00459 and includes both a positive reference, Rabbit IgG in red, and a negative reference, MBP in blue. The boxes encompass 50% of the values, whereas the whiskers enclose 98% of the data. The median is represented by a horizontal line, while the mean is displayed as a small rectangle. Several regions may be identified with signal intensities significantly above the remaining peptides and the negative control. These include peptides 50, 51, 59, 60, 79, 80, and 81. The intensities of these peptides varied from 1200 A.U. for peptide 81 to roughly 6000 A.U. for peptide 59 which albeit significantly above 500 A.U. scored by the negative reference are far off from the 30000 A.U. achieved by the positive reference.

**Figure 4:**
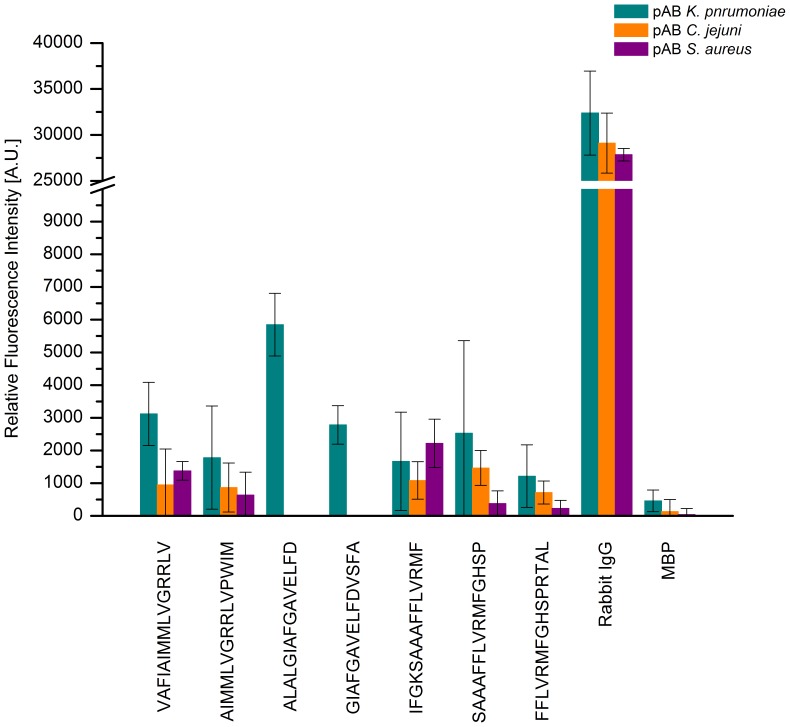
Specificity binding analysis of epitope peptides of KPN_00459. After incubation with different antibodies reactive to to *K. pneumoniae* (green), *C. jejuni* (orange), and *S. aureus* (purple), peptides 59 (ALALGIAFGAVELFD) and 60 (GIAFGAVELFDVSFA) displayed mean signal intensities of 6000 and 3000 A.U. respectively for *K. pneumoniae* antibody incubation. Contrastingly, the mean signal intensities of these two peptides dropped to less than 500 A.U. after incubation with the other antibodies.

For the putative transport protein, KPN_00459, three regions scored intensities above 1000 A.U. including peptides 50 and 51, 59 and 60 as well as 79–81, see Figure 3. The positive reference again reached a mean value above 30000 A.U., while the negative control levelled out at less than 500 A.U. After specificity assays, the peptides 59 and 60 revealed predominantly specific binding by the *K. pneumoniae* antibody as the mean values dropped from approximately 5800 and 2800 A.U. respectively, to less than zero for all other antibodies tested, see Figure 4. Thus, a consensus for this epitope can be derived to GIAFGAVELFD. Contrastingly, the remaining peptides revealed equal intensities independent of the antibody used.

**Figure 5:**
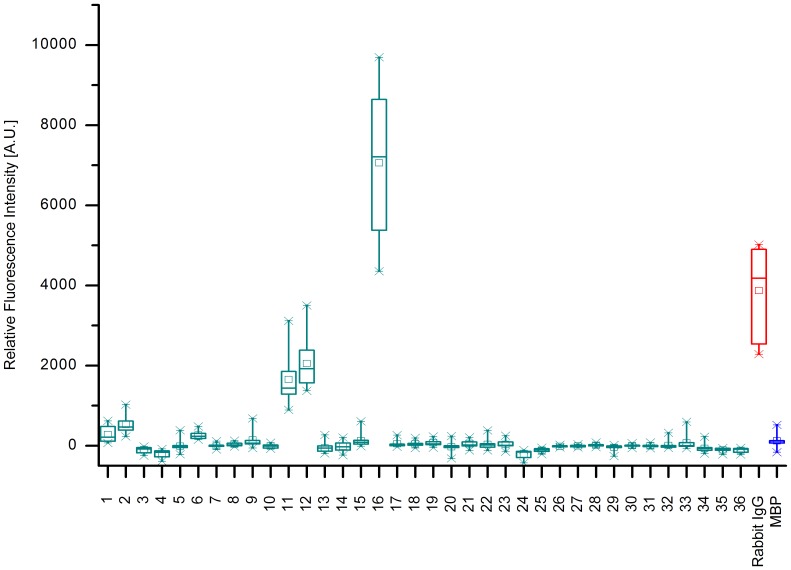
Epitope Mapping of KPN_00466. Box-whisker plot (n = 10) displaying the resulting relative fluorescence intensities of the overlapping peptides. As controls, Rabbit IgG (red) and MBP (blue) are indicated. Each box represents 50% of the values, while the whiskers entail 98% of the data. The median is represented by a horizontal line and the mean displayed as a small rectangle. Peptides 11 and 12 are the only two adjacent peptides reaching intensities of 2000 A.U. within close proximity of the positive control at 4000 A.U indicative of a potential linear epitope. All the remaining peptides fell significantly short of these values except for peptide 16 which topped all other intensities by peaking at approximately 7000 A.U. However, as neither peptide 15 nor 17 showed any elevated intensities, the presence of a linear epitope at position 16 is rather unlikely.

**Figure 6:**
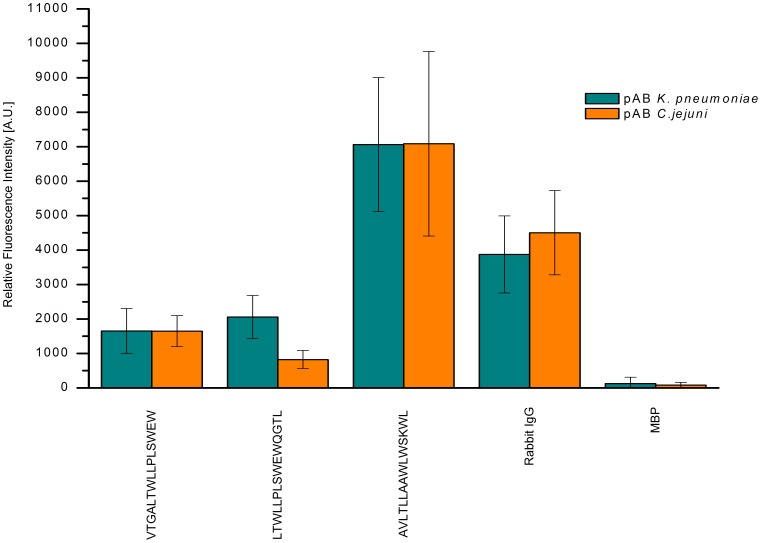
Specificity binding analysis of epitope peptides of KPN_00466. Bar chart representing the mean relative fluorescence intensities (n = 10) of each peptide potentially harboring a linear epitope site after incubation with polyclonal antibodies reactive to *K. pneumoniae* (green) and *C. jejuni* (orange). None of the peptides shows a peculiar specific interaction; rather signal intensities are in the same vicinity for each peptide independent of the antibody used. This indicates mainly non-specific binding to occur.

Finally, for the third protein, KPN_00466, one pair of adjacent peptides, namely 11 and 12, achieved mean values of approximately 2000 A.U. within close proximity to the positive reference, as depicted in Figure 5. Additionally, peptide 16 displayed the highest overall mean value with more than 7000 A.U., however neither of the two neighboring peptides (15 and 17) attained values of any significance. Thus, the presence of a linear epitope in that region is rather unlikely. Besides, as specificity assays illustrated, none of the three peptides showed specific interaction to the *K. pneumoniae* antibodies alone, see Figure 6. Rather, signal intensities with antibodies reactive to *C. jejuni* fell into the same scope. Therefore, nonspecific binding to these peptides is probable. The remaining three proteins under investigation failed to disclose any linear peptide region with significant signal intensities to assume linear epitopes to exist.

### Epitope accessibility and, homology

**Figure 7:**
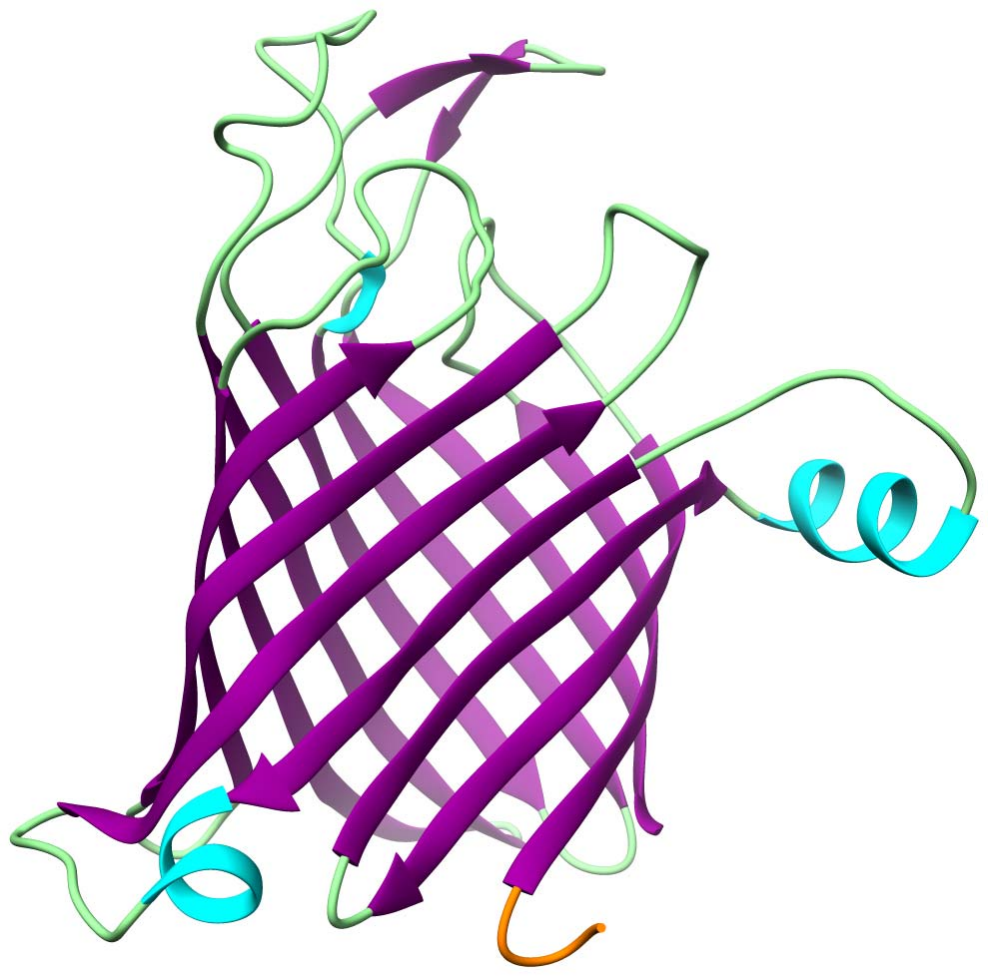
Partial 3d model of KPN_00363. The model incorporates residues 31 to 294 and thus spans most of the protein except for the first 30 residues. Coils are displayed in green, helices in light blue and strands in purple. The N-terminal region of the model, i.e. starting with amino acid 31, is highlighted in orange. The model was based on the bacterial nucleoside transporter Tsx of *E. coli*. A major feature of the given model is the prominent beta barrel structure that originates from the abundance of beta strands. This is a typical feature of transport and channel proteins spanning the outer bacterial membrane. Contrastingly, the identified linear epitope GAVVALSTTFA is located at the very beginning of the protein and thus not included in the given model. However, it is likely an extension of the truncated N-terminal region marked in orange.

For a better understanding of the suitability of the identified linear epitopes, structural modelling was employed using the SWISS Model automated mode. For KPN_00363 a model was constructed based on the crystal structure of the bacterial nucleoside transporter Tsx of *E. coli*. However, the derived model only spans residues 31 to 294 and as such does not contain the derived consensus sequence of the linear epitope GAVVALSTTFA. Nevertheless, the model displays a beta barrel structure typical of outer membrane-spanning transport proteins, see Figure 7 for details. The first residues of the derived model, starting at position 31, are marked in orange and present a coiled region outside of the beta barrel. Therefore, the likelihood of the GAVVALSTTFA region to reside within the barrel is slim. Rather, an extension of the truncated coil seems plausible. Consequently, the potential accessibility of the identified linear epitope appears high.

**Figure 8:**
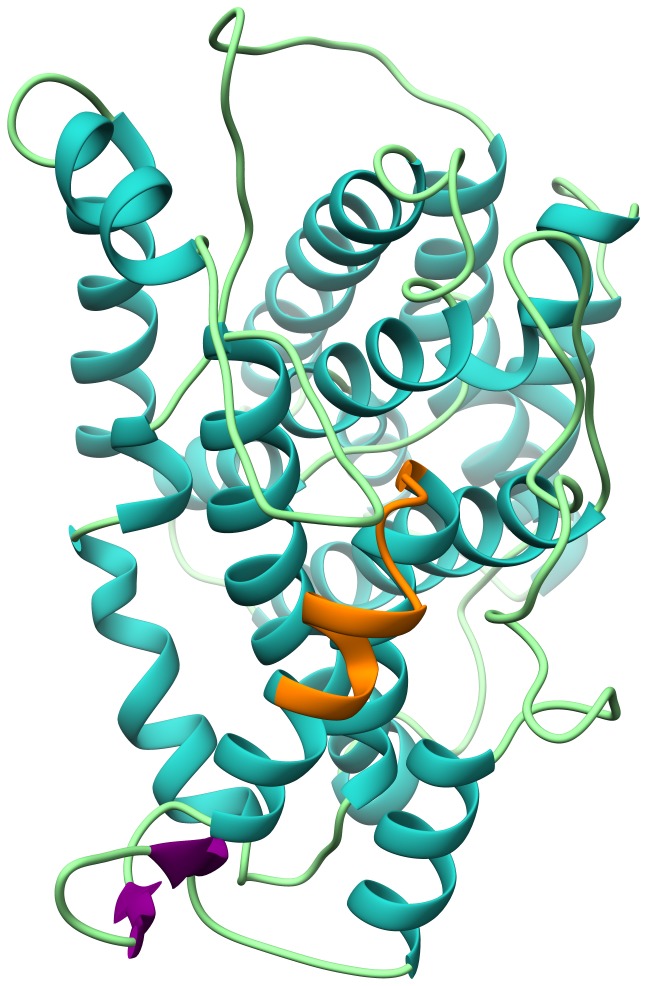
3d model of predicted structure of KPN_00459. The model was predicted using the automated mode of the SWISS MODEL application by Expasy (University Basel). As a template the crystal structure of a NA(+)/H(+) antiporter NhaA of *E. coli* was used. The resulting model spans residues 12 to 390 of the full-length protein and was subsequently dyed using the Chimera 1.7 software. Coils are depicted in light green, beta strands in purple and alpha helices in blue. The potential linear epitope GIAFGAVELFD is highlighted in orange. It comprises part of an alpha helix, a connective coil and the start of the next alpha helix.

Furthermore, the predicted 3d structure of the first part of KPN_00459 is displayed in Figure 8. Contrary to KPN_00363, two models were devised for KPN_00459. However, only one encompasses the identified linear epitope GIAFGAVELFD, which is highlighted in orange and is situated as part of an alpha helix and an adjacent loop. As the sequence is not enclosed by other residues or structures, good accessibility ought to be provided.

**Figure 9:**
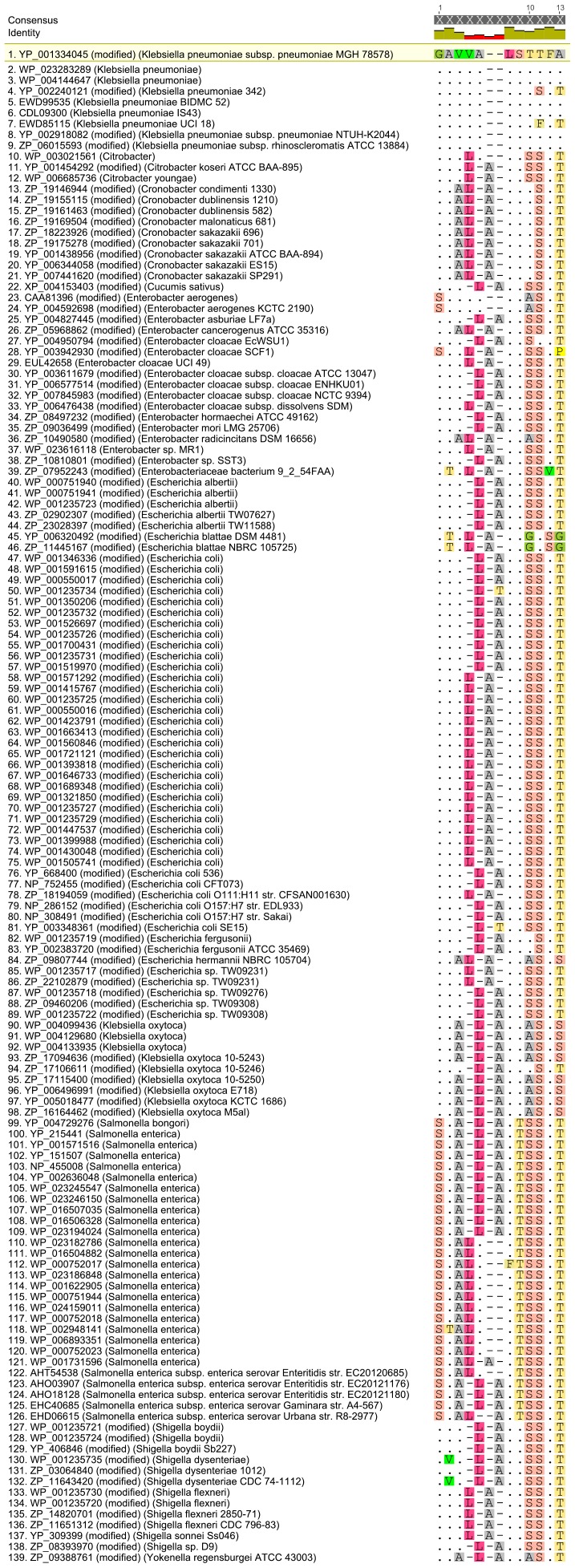
Homology of linear epitope sequence GAVVALSTFFA of KPN_00363. The sequence derived from K. pneumoniae MGH 78578 was used as a reference. Identical residues are marked by dots, gaps by a horizontal dash and differences by the single letter amino acid code. Seven of nine *K. pneumoniae* strains show identical epitope sequences, while two strains display changes in two residues. Threonine replaces alanine at position 11, a change observed not only in these two strains but in almost all other bacteria within the list. Additionally, threonine at position 9 is substituted by either serine or phenylalanine. Bacteria other than *K. pneumoniae* show an additional number of amino acid substitutions, most prominently leucine for valine at position 4 and serine for threonine at position 8. In *S.enterica* the changes become more pronounced. Glycine at position 1 is replaced by serine, valine at position 3 replaced by alanine and threonine inserted for serine at position 7. In some rare cases, other residues have also been substituted, e.g. valine replaces alanine at position 2 in *Shigella dysenteriae*.

In order to predict the potential specificity of the epitopes, homology analyses were performed. Whereas GIAFGAVELFD of KPN_00459 exhibits a broad homology throughout with all residues identical in closely related species, see Figure S3: homology of KPN_00459, GAVVALSTTFA of KPN_00363 features four residues likely specific for *K. pneumoniae* within this particular sequence. The variance of the latter epitope's sequence considering closely related species to *K. pneumoniae* is summarized in Figure 9. Specifically, the following residues have been replaced: valine by leucine (position four), both threonine residues by serine residues (position 8 and 9) as well as alanine by threonine (position 11).

### Alanine Scan of GAVVALSTTFA

**Figure 10:**
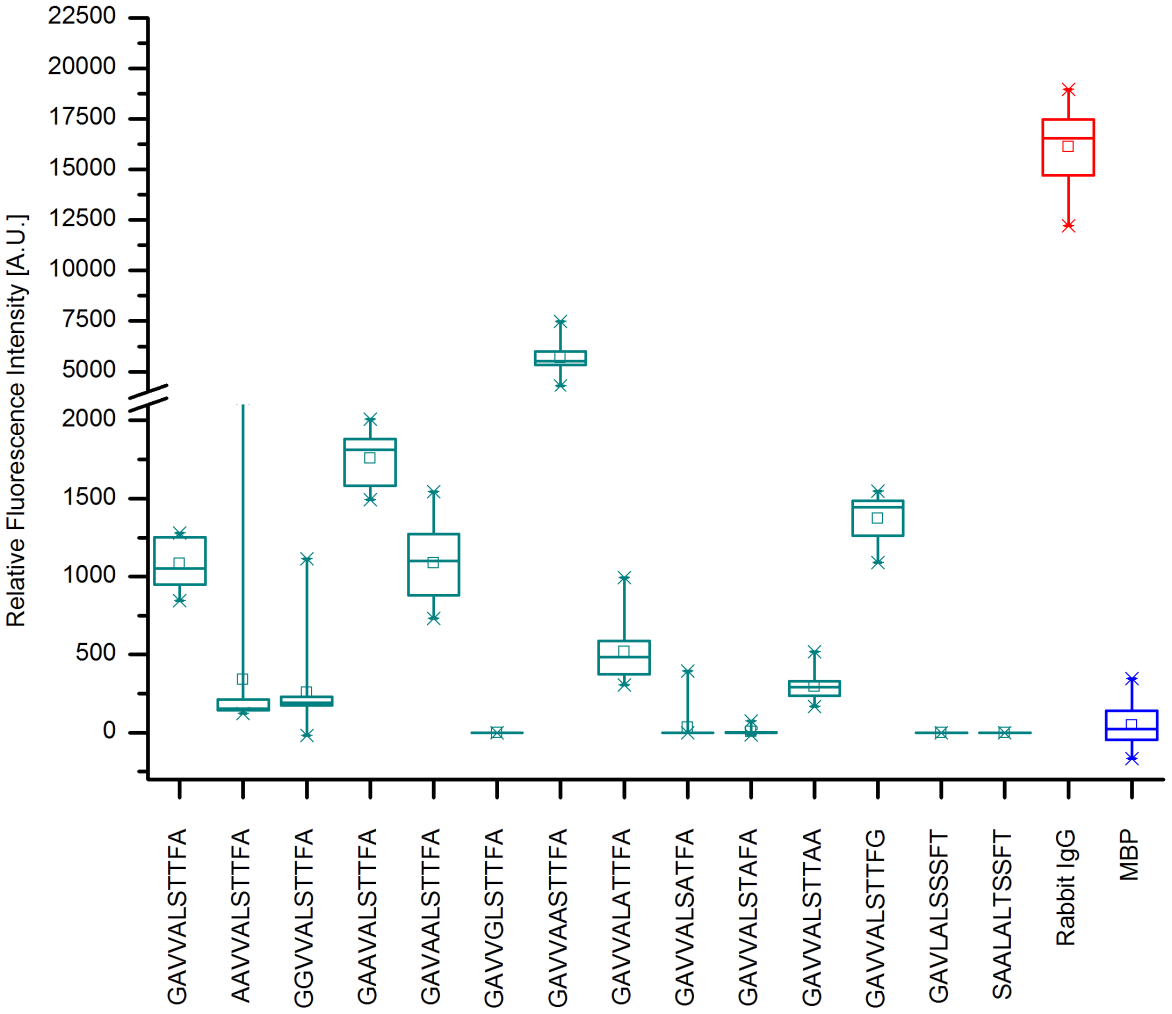
Alanine scan of GAVVALSTTFA of KPN_00363. Box-whisker plot (n = 12) of GAVVALSTTFA after alanine/glycine scanning. The box comprises 50% of the data, while the whiskers enclose 98%. The median is represented by a small horizontal line and the mean by a small rectangle. Rabbit IgG served as a positive reference, whereas MBP was used as a negative control. If alanine was present in the original present it was replaced by glycine, otherwise each amino acid was stepwise replaced by alanine. Additionally, GAVLALSSSFT and SAALALTSSFT were included as they resemble sequences present in *E. coli* and *S. enterica*. Switching glycine (position 1), alanine (positions 2 or 5), or threonine (positions 8 and 9) to alanine or glycine, results in a significant drop in signal intensities to levels below or at the negative control. In contrast, substituting valine (position 3) or leucine (position 6) by alanine, leads to an increase in signal intensities to 1700 A.U. and more than 5600 A.U., respectively. Note the different axis scales prior and after axis break at 2000 A.U.

**Figure 11:**
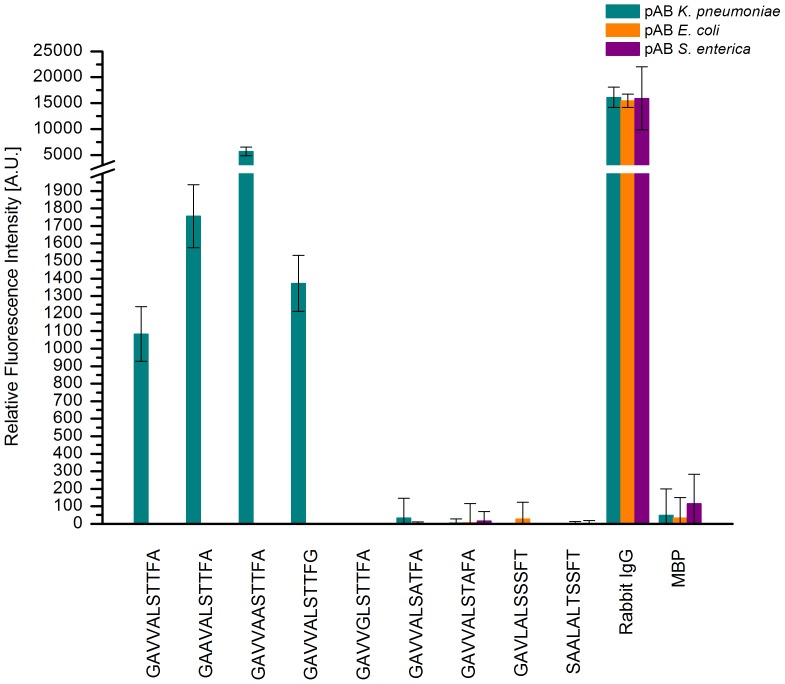
Specificity assay of GAVVALSTTFA and derivatives. The bar chart represents the mean signal intensities (n = 12) of GAVVALSTTFA and several modified peptides with single amino acid replacements incubated with antibodies reactive to *K. pneumoniae* (green), *E. coli* (orange) or *S. enterica* (purple). The sequences on the left represent the original epitope and modified versions displaying an increase in signal intensity for *K. pneumoniae* antibodies. In contrast, sequences on the right harbor modifications causing a significant drop in intensity for *K. pneumoniae*. Rabbit IgG is used as a positive reference and MBP serves as a negative control. None of the sequences tested displayed any significant signal intensity above the negative control when incubated with either *E. coli* or *S. enterica* antibodies.

The influence of sequence variations on the binding capacity of the identified epitope GAVVALSTTFA was subsequently examined by performing an alanine scan. Its results are summarized in Figure 10. The original consensus epitope sequence shows a mean intensity of approximately 1000 A.U. Alterations of the first, second, fifth, eighth and ninth residue lead to a significant drop of signal intensities. Consequently, signals of less than 300 A.U., in close proximity to the negative control MBP with 50 A.U. are obtained. Contrastingly, by altering residues three or six of the consensus sequence GAVVALSTTFA, i.e. replacing the first valine or leucine by alanine, the resulting mean signal intensities are significantly increased to more than 1700 A.U. for replacing valine and surpassing 5600 A.U. after changing leucine to alanine. Additionally, specificity assays showed no significant signal intensity for antibodies reactive to closely related species, i.e. *E. coli* and *S. enterica*. Incubation with antibodies reactive to either of those two bacterial species resulted in signal intensities in the neighbourhood of the negative control independent of sequence alterations, see Figure 11. This was also true for GAVLALSSSFT and SAALALTSSFT.

## Discussion

The screening of a cDNA based expression library of *K. pneumoniae* has successfully identified a number of novel, previously unknown immunogenic proteins. This is well in accordance to previous achievements of this method, which we have employed for the identification of both *C. jejuni* [5] and *S. enterica* [6] antigens. Consequently, in this current study we aimed to detect suitable proteins for the specific identification of *K. pneumoniae*. Furthermore, identification of specific antigens might improve treatment opportunities including the development of suitable vaccines [20]. Finally, detecting proteins with yet unknown functional and structural information to be antigenic might be an indication of their potential involvement in the pathogenic nature of an organism. Thus, these proteins might be suitable access points for future investigations to improve the understanding of the underlying pathogenicity and virulence of *K. pneumoniae*.

Within the 14 proteins only a subset was selected for further analysis using epitope mapping. The rationale for these selections was based on three distinct features: the resulting Q value, homology as well as functional and structural properties. While the Q value mirrors a normalized intensity compared to a positive reference, it does not fully account for differences in expression levels, misfolding and other factors which might have had an influence on the binding reaction and thus on the overall intensity. Still, it facilitates to rank the proteins and increases the likelihood of a given protein to be immunogenic if its Q value is significantly high. However, proteins with outstanding Q values were not chosen for epitope mapping by default. Rather, the known homology and corresponding distribution of each protein was carefully evaluated. Thus, proteins like KPN_02199 and KPN_03668 albeit scoring high Q values were exempt from epitope mapping as they display a broad spectrum of homologous proteins across bacterial species.

Finally, the intrinsic properties of each protein, if available, were closely scrutinized.

Therefore, proteins with hypothetical character were predominantly chosen, as information on them is confined. In addition, all types of membrane-associated proteins deserved a better look, as these type of proteins offer a more direct route and accessibility, which might be of utmost importance in a future rapid point-of-care device detecting whole organisms.

After epitope mapping, three of the six proteins under investigation revealed sites with potential linear epitopes. Still, the remaining proteins displayed no such sequences. This is, however, in accordance to the general prevalence of structural epitopes in comparison with linear epitopes in nature. In fact, approximately 90% of all epitopes are conformational rather than sequential [21], [22]. Despite these potential shortcomings of the used linear epitope mapping method, a number of intriguing linear epitopes have been identified. Notably, three proteins, KPN_00363, KPN_00459 and KPN_00466, harbored sites with potential linear epitopes. Further careful examination, however, excluded KPN_00466 from future applications as the identified epitope sequence were nonspecific for *K. pneumoniae*, which might be due to the conserved character of the protein within the family Enterobacteriaceae. Still, KPN_00466 is a membrane protein and upcoming investigations might help to elucidate applications using this protein either for prevention of *K. pneumoniae* infections or detection thereof.

In contrast, the other two proteins displaying linear epitopes, KPN_00363 and KPN_00459, indicated some specificity with the antibodies tested and two linear consensus sequences could be derived, GAVVALSTTFA and GIAFGAVELFD, respectively. While the former sequence is present in KPN_00363, an ion channel protein Tsx, which is conserved among Enterobacteriaceae, the latter sequence is part of KPN_00459, a hypothetical protein with high similarity to a cation:proton antiport protein and conserved among Proteobacteria. Consequently, the homology of GIAFGAVELFD (KPN_00459) is high throughout different bacterial species, which renders the sequence inapt for specific *K. pneumoniae* detection and other applications. In contrast, GAVVALSTTFA (KPN_00363) displays alterations within this sequence for bacterial species other than *K. pneumoniae*. As small as these alterations appear, they appear to have a crucial effect on the binding of antibodies to the target sequence and thus benefit specificity. This is well in line with the experimental results that indicate no binding by antibodies reactive to other bacteria to this sequence or any of its alterations. Moreover, alanine scanning revealed a number of residues to be paramount for antibody binding. Consequently, replacing the glycine (position 1), alanine (position 2), alanine (position 5) or either threonine (positions 7 and 8) leads to a significant loss of antibody binding observable by a dramatic reduction in signal intensity. On the contrary, removing either the first valine or leucine and inserting an alanine residue as a replacement results in a significant increase in signal intensity, potentially hinting at an improved antibody binding. The reason for these effects remains nebulous; however, it is not caused by a simple change in secondary structure. This is apparent as both AAVVALSTTFA and GAVVAASTTFA, two sequences at opposite sides regarding signal intensity, are predicted to consist solely of an alpha-helix as compared to the original sequence, which is predicted to contain a beta-strand (position 1 to 7) and a helical part (position 8 to 11) by EMBOSS garnier. Furthermore, as the original sequence is fully constructed of uncharged amino acids, alterations via alanine or glycine do not change the overall charge of the peptide. Consequently, the differences in signal intensity cannot be deduced to implementation or removal of charged residues. Finally, changes in hydrophobicity albeit present are minimal at best and may not suffice to explain the observed characteristics. Still, one has to bear in mind that the accuracy of prediction based tools is often lacking, especially for EMBOSS garnier with an accuracy in the range of 65%. Consequently, some of the predicted secondary structures might differ. Despite some uncertainty as to the mechanistic cause of the altered peptides to behave as they did, one fact remains obvious. None of the peptides showed any significant binding to antibodies not raised against *K. pneumoniae*. Therefore, after alanine scanning, GAVVALSTTFA remains a suitable candidate for specific *K. pneumoniae* detection or treatment. Still, accessibility of the epitope is paramount to a quick diagnostic tool. Thus, modelling of the 3d structure of the protein was performed. Unfortunately, the 3d modelling only succeeded for the part of the protein, which does not contain said linear epitope sequence. Nevertheless, the pronounced beta barrel structure of the channel protein is visible and the linear epitope sequence, albeit absent, likely to be an extension of the freely accessible N-terminal region outside the barrel structure. Thus, accessibility of the epitope by an antibody might be pronounced without the need to enter the cell or channel. Membrane proteins harboring a beta barrel like structure have been shown in bacteria to be exclusively found in the outer membrane [23]. Moreover, the mobile coils on the extracellular sides of these membrane proteins are pivotal for their function or interaction with other molecules. This renders the identified sequence an intriguing target for antibody-based detection. Additionally, channel proteins harboring beta barrel like structures have been shown to be immunodominant in other bacterial species such as *Salmonella, Haemophilus influenzae, E. coli, Neisseria meningitides, Shigella dysenteriae* and *Chlamydia trachomatis* [24]–[29].

On the contrary, the model for KPN_00459 encompasses the identified consensus sequence of the linear epitope GIAFGAVELFD, which is part of a loop between two alpha helices. The abundance of alpha helices suggests the protein to span the inner membrane [23]. In this topological design, helices are mostly located within the membrane, notably as transmembrane domains, whereas loops are located either on the cytoplasmic or periplasmic side of the cell.

When considering prediction based methods, such as S_TMHMM for topological domains [30], [31] or EMBOSS antigenic [32], [33], part of GIAFGAVELFD is assumed to be extracellular. Combined with the high flexibility and degrees of freedom of random coil structures, the likelihood for good accessibility is high rendering the sequence a potentially attractive target for whole cell detection despite its lack of specificity. Furthermore, these findings support the 3d model and underline the accuracy of the specified structure.

Another key aspect in determining the accuracy of the 3d model prediction is a so-called Z-score [34]. The Z-score for the model of KPN_00363 is −4.285 and −8.895 for KPN_00459 respectively. Although the values are significantly below zero that does not inevitably indicate models of poor accuracy. In fact, low Z-scores are often obtained if the protein under investigation is membrane-associated. This is mainly due to the inverse physicochemical properties of membrane proteins in comparison to soluble ones. Hence, the low Z-scores are more likely induced by this effect than caused by an insufficient accuracy of the models.

In conclusion, we have successfully identified several novel antigens of *K. pneumoniae* and identified three proteins potentially harboring linear epitopes. Subsequently, we achieved to identify two sequences displaying specificity during experimental investigations; however, one of these is doubtful as homology analysis has revealed it to be highly conserved among a broad spectrum of bacterial species. Still, GAVVALSTTFA of KPN_00363 was identified to be specific both experimentally and has shown four residues within the eleven amino acid sequence to occur predominantly in *K. pneumoniae* only. Thus, the likelihood for this linear epitope to be specific is high. This assumption was confirmed by alanine scanning revealing a number of pivotal residues for antibody binding. Moreover, it was unearthed that neither *E. coli* nor *S. enterica* antibodies were able to bind to any of the sequences, original and modified. Subsequent investigations might help to further nurture the insight into the suitability of this peptide for diagnostic and therapeutic applications. Thus, monoclonal antibodies ought to be devised to be used for affinity investigations via BIAcore and to determine kinetics. Furthermore, monoclonal antibodies could be used within a potential diagnostic tool and after validation ought to be tested with whole bacteria. If these antibodies are able to specifically detect intact *K. pneumoniae* cells, the resulting antibody might well be suited for integration into a point-of-care device.

In a different approach, the identified epitope sequence could easily be produced in large quantity. This peptide might serve some role in serological screenings, especially if it proves to be immunodominant. Consequently, antibodies against this epitope might be present in a plethora of patient sera.

Finally, all proteins identified here might be suitable candidates for vaccine development independent of the existence of a linear epitope, as structural epitopes might well be present and antigenicity ensured. Nevertheless, additional in-depth analysis is required to determine a number of the key aspects of vaccine development prior to use.

## Methods

### Bacterial strain

As a donor of RNA the fully-sequenced strain *K. pneumoniae* MGH78578 was grown on solid Trypticase-Soy-Agar (TSA) for 24 h at 37°C under aerobic conditions. For RNA isolation a liquid culture was prepared by inoculating 10 ml of brain-heart-infusion broth (BHI) with a single colony and incubated overnight at 37°C, 140 rpm. This overnight culture was used to inoculate a flask containing 100 ml fresh BHI medium. The cells were harvested 6 h after inoculation.

### Antibodies

For initial screening a Rabbit polyclonal IgG antibody to *K. pneumoniae* (Acris AP00792PU-N) was used. For further microarray analyses of a subset of candidate proteins, ELISA and epitope mapping this antibody was used as well as Rabbit polyclonal IgG antibody to *K. pneumoniae* (Abcam ab20947). The antibodies were generated with *K. pneumoniae* ATCC 43816 serving as an immunogen. Specificity assays were performed employing rabbit polyclonal IgG antibody to *C. jejuni* (Acris AP24002PU-N), *S. aureus* (Fitzgerald 20C-CR1274RP), *E. coli* (Abcam ab137967) and *S. enterica* (Abcam ab35156). Detection was achieved by usage of secondary antibodies. Goat polyclonal antibody to Rabbit IgG conjugated with Chromeo-546 (Abcam ab60317) for fluorescent and antibody conjugated with Horseradish peroxidase (Abcam ab6721) for a colorimetric readout were applied where appropriate.

### Harvesting of cells, lysis and total RNA extraction

The cells were harvested by centrifugation (2000×g, 10 min) and the resulting supernatant discarded. The pellets were resuspended in fresh BHI medium. For stabilisation of the RNA, 1 ml of RNAprotect Bacteria Reagent (Qiagen) was added to 0.5 ml of bacterial suspension and processed according to the manufacturer's instructions. Lysis was performed with 200 µl of lysis buffer (30 mM Tris-Cl, 1 mM EDTA, 15 mg/ml Lysozyme, >12 mAU Proteinase K) by pipetting and vortexing for 10 s. After incubation, 700 µl buffer RLT and 500 µl 96% Ethanol were added and the lysate applied to RNeasy Bacteria Mini Kit spin columns (Qiagen) for RNA isolation following the manufacturer's instructions. During this procedure an on-column DNase digest was performed using RNase-free DNase I solution (Qiagen) according to the manufacturer's instructions. The isolated total RNA was eluted in 50 µl of RNase-free water and its concentration and purity analyzed by Nanodrop (Peqlab) measurements.

### Analysis of RNA integrity

The quality of isolated RNA was assessed using the RNA 6000 pico kit and Bioanalyzer 2100 (Agilent). The total RNA was diluted to a working concentration of 200–500 pg/µl. The analysis was performed following manufacturer's instructions and the RNA integrity number (RIN) calculated by the 2100 Expert software (Agilent). The RIN is defined to fall into a range of 0 to 10, with a higher score indicating a more intact RNA, whereas lower numbers are associated with degraded RNA molecules [35].

### Polyadenylation of total RNA

In order to use bacterial mRNA as a substrate in cDNA synthesis, polyadenylation was mandatory. The tailing was achieved using the Poly(A) Polymerase tailing kit (Epicentre) following the alternate protocol offered by the manufacturer. Briefly, up to 10 µg of total RNA were combined with 2 µl Poly(A) Polymerase reaction buffer, 2 µl 10 mM ATP, 0.5 µl Riboguard RNase Inhibitor and 1 µl Poly(A) Polymerase (4 U) in a total reaction volume of 20 µl. The reaction was incubated for 20 min at 37°C, terminated by the addition of 1 µl 0.5 M EDTA and purified by RNeasy Mini Kit (Qiagen) following manufacturer's instructions. Yield and purity were determined by nanodrop measurements.

### cDNA synthesis

For cDNA synthesis the In-fusion SMARTer Directional cDNA Library Construction Kit (Clontech) was used according to manufacturer's instructions with slight modifications. 3.5 µl total, polyadenylated RNA were mixed with 1 µl of 3′ In-Fusion SMARTer CDS Primer, heated first for 3 min at 72°C and then incubated for additional 2 min at 42°C. After addition of 5.5 µl Mastermix (2 µl 5x First Strand Buffer, 0.25 µl 100 mM DTT, 1 µl 10 mM dNTPs, 1 µl 12 µM SMARTer V Oligonucleotide, 0.25 µl RNase Inhibitor and 1 µl SMARTscribe Reverse Transcriptase) the tubes were incubated for 90 min at 42°C. The reaction was terminated at 68°C for 10 min.

For second strand cDNA synthesis two 2 µl aliquots of first strand reaction were used in long distance PCR using Phusion Polymerase (Finnzymes). Each PCR reaction was comprised as follows: 2 µl First-strand reaction, 70 µl RNase-free water, 20 µl 5x Phusion HF buffer and 2 µl each of dNTP mix (10 mM), 5′ PCR Primer II A (12 µM), 3′ In-Fusion SMARTer PCR Primer (12 µM) and Phusion Polymerase with a total reaction volume of 100 µl. The PCR reactions were subjected to the cycling program with 98°C for 1 min as initial denaturation followed by 15 cycles of 10 s denaturation at 98°C, 30 s of primer annealing at 65°C and 6 min extension at 72°C. For improved PCR results optimization was performed as follows; 30 µl of the 15 cycle experimental tube were transferred to a separate PCR tube, cycling commenced and aliquots of 5 µl each were collected after 15, 18, 21, 24 and 27 cycles total. The different cycles were compared by gel electrophoresis and the experimental tubes subjected to additional cycles if necessary. Finally, PCR reactions were purified using the QIAquick PCR Purification Kit (Qiagen). The purity and yield of each reaction were analyzed by nanodrop measurements.

### Normalization of cDNA

Normalization of double-stranded cDNA was achieved with the Trimmer-2 cDNA Normalization Kit (Evrogen) to reduce the number of cDNA molecules derived from rRNAs. Briefly, 12 µl of cDNA (approx. 100 ng/µl) were mixed with 4 µl of 4x Hybridization buffer. For the trimming reaction 4 µl of this mixture were distributed to four different PCR tubes and overlaid with a drop of PCR-grade mineral oil. After centrifugation (13000×g, 2 min), the tubes were incubated for 2 min at 98°C followed by 5 h at 68°C. Next, pre-heated (68°C) Duplex-specific nuclease (DSN) master buffer was added to each tube and incubation prolonged for 10 min. DSN was added to the first three tubes in decreasing concentrations – 1 U/µl, 0.5 U/µl and 0.25 U/µl – with the fourth tube receiving DSN storage buffer and no enzyme as a control reaction. The incubation prolonged for 25 min at 68°C. After addition of 5 µl DSN stop solution and subsequent incubation for 5 min at 68°C, the tubes were placed on ice. The chilled reaction was diluted by addition of 25 µl sterile, RNase-free water. For amplification of normalized cDNA, 1 µl of each reaction was used as template in PCR. Each PCR reaction contained 1 µl of template from the normalization reaction, 33 µl nuclease-free water, 10 µl 5x Phusion HF buffer, 1 µl 10 mM dNTP mix (NEB), 2 µl of each primer 5′ PCR Primer II A (12 µM), 3′ In-Fusion SMARTer PCR Primer (12 µM) and 1 µl Phusion Polymerase. The PCR was performed with initial denaturation at 98°C for 1 min and seven cycles of denaturation at 98°C for 10 s, primer annealing at 65°C for 30 s and extension at 72°C for 3 min, respectively. For optimization, the control tube was subjected to 7, 9, 11, and 13 cycles with 12 µl aliquots taken every two cycles. The optimization samples were analyzed by gel electrophoresis (1% agarose, TAE, 100 V) and the optimal cycle number determined. The remaining three tubes were subjected to 9+ X cycles with X being the differential of the optimized cycles to the originally performed seven cycles. After the second PCR, the experimental reactions were compared to the optimal control reaction using gel electrophoresis as above. Reactions showing a successful normalization were combined and used in a third PCR reaction. After the final PCR, the reactions were purified by QIAquick PCR Purification Kit.

### In-fusion cloning and cloning vector

For cloning pFN18A (Promega) was used as a vector, as it features a N-terminal encoded HaloTag fusion protein, which allows for specific and covalent binding to a unique ligand, thus reducing background and minimizing cross-reactivity in immunoassays with HaloLink microarrays harboring the ligand on its surface. First, the vector needed to be linearized to be used with the In-Fusion cloning technology. This was achieved by reverse PCR using IFS 18A for (5′ TTGATACCACTGCTTTTCCATGGCGATCGCGTTATC 3′) and IFS 18A rev (5′ TCTCATCGTACCCCGTGTTTAAACGAATTCGGGCTCG 3′). Each reaction contained 2 µl each of 1∶10 diluted pFN18A (10 ng/µl) and the two primers, 10 µl 5x Phusion HF buffer, 1 µl 10 mM dNTPs, 0.5 µl Phusion Polymerase and 32.5 µl nuclease-free water to reach a total reaction volume of 50 µl. The PCR was run using a 25 cycle two-step program with 98°C denaturation for 10 s and 4 min extension at 72°C. After completion, 2 µl of *Dpn*I (20 U/µl) were added to the reaction and incubated at 37°C for 1 h. The presence of a single band was checked by gel electrophoresis and the remaining reaction purified by QIAquick PCR Purification Kit. Cloning of normalized cDNA and linearized pFN18A vector was performed following the manufacturer's instructions within the In-Fusion SMARTer Directional cDNA library construction kit (Clontech).

### Electroporation

2 µl of the cloning reaction were mixed with 25 µl of electrocompetent Acella *E.coli* cells (Mobitec), a BL21 derivative, and electroporated in 1 mm cuvettes using the EasyjecT Plus electroporator (Peqlab). Conditions for electroporation were as follows: voltage = 1400 V, capacity = 25 µF, resistance = 200 Ω and a pulse duration of 5 ms.

The electroporated cells were added to 970 ml of Super Optimal Broth with Catabolite Expression (SOC) and incubated at 37°C for 1 h with shaking at 250 rpm. Afterwards, 150 µl of the transformation reaction were plated on Lysogeny Broth (LB) Agar containing ampicillin. For each reaction at least two plates were prepared and incubated at 37°C for 16 h.

### Selection of clones for screening, protein expression, lysis

A total number of 1536 clones were selected and transferred to 1.3 ml U96 DeepWell Plates (Nunc) containing 0.8 ml LB-amp. The plates were incubated overnight at 37°C, 130 rpm. On the next day, the DeepWell Plates were centrifuged, the supernatant discarded and the pellets resuspended in 370 µl of LB-amp. A new set of U96 DeepWell Plates was prepared with 850 µl of fresh LB-amp and inoculated with 100 µl each from the resuspended overnight cultures. The remaining 270 µl of resuspended overnight culture were mixed with 30 µl of sterile-filtered DMSO and stored at −80°C. The newly inoculated plates were incubated for 6 h at 37°C, 130 rpm. Afterwards, the temperature was reduced to 20°C, incubation continued for 1 h and protein expression induced by addition of 2 µl of 0.5 M β-D-1-thiogalactopyranoside (IPTG). Incubation persisted overnight at 20°C, 130 rpm. The cells were harvested by centrifugation (2500×g, 10 min), the supernatant discarded and the pellets frozen at −20°C. After 15 min the pellets were resuspended in 180 µl of EasyLyse Bacterial Protein Extraction Solution (Epicentre) and incubated for 5 min at room temperature. DNase I was mixed with DNase reaction buffer (10 mM Tris-HCl, 2.5 mM MgCl_2_, 10 mM CaCl_2_), added to the reaction and incubation was carried on for 10 min at 37°C. The plates were centrifuged to collect cell debris for 3 min at 2500×g. For each sample 10 µl of lysate were transferred to 384 microtiterplates (Genetixx), which were used as reservoirs for the spotting procedure.

### Immunoscreening using Halolink Microarrays

The samples were spotted onto HaloLink slides (Promega) using the QArray2 microarray spotter (Molecular Devices). 384 different samples were spotted per slide with three replicate slides per screening. In total 1536 samples were screened on 12 slides (n = 3). Each sample was spotted as quadruplicates with controls in two identical sets of eighteen 10×10 subarrays each (total number of spots per slide 3600). The controls used included HT-OmpA and HT-OmpF as positive reference proteins as these have been described as immunodominant before. As specificity controls HT-ArgC and HT-PyrC were used, representing proteins without known immunodominant behaviour, thus binding of the polyclonal antibodies is not expected. In addition two different *E.coli* strains – Acella electrocompetent cells and KRX single-step competent cells (Promega) – were spotted as further controls. As those two lack proteins expressed with a HaloTag, they are used as negative controls.

After spotting of the samples, the slides were incubated for 1 h at room temperature in a humidity chamber. Next, slides were washed with PBS+0.05% IGEPAL CA-630 (PBSI, Sigma Aldrich) and dried by nitrogen flow. The 2 Well ProPlate module (Grace Biolabs) was attached to each slide. The top chamber was filled with 1.5 ml of rabbit-polyclonal antibody to *K. pneumoniae* (Acris, 2 µg/ml) in PBS. The bottom chamber was incubated with PBS only. After 2 h of incubation at room temperature with gentle rocking, both chambers of each slide were washed three times with 2 ml of PBSI. Secondary antibody (Goat-polyclonal to Rabbit IgG conjugated with Chromeo-546, Abcam, 5 µg/ml) was subjected to each chamber in PBS and the slides were incubated at room temperature for 2 h in the dark under gentle rocking. Finally, slides were washed three times with PBSI, the ProPlate modules removed and the slides dried by nitrogen flow. The slides were scanned on an Axon Genepix 4200A laser scanner (Molecular Devices) with the following settings: 532 nm laser, PMT gain 400, 40% laser power, lines to average 1, 10 µm resolution and standard green emission filter at 575 nm.

### Microarray Analysis

The raw data sets of all the microarray analyses in this publication have been deposited in NCBI's Gene Expression Omnibus [36] and are accessible through GEO Series accession numbers GSE52536, GSE52537, GSE52538, and GSE60588. The median fluorescence intensity of each spot corrected by the local background (median F532 – B532) was used. Further, relative fluorescence intensity (rfi) was calculated by subtracting the signals of the bottom chamber from the raw data signals of the top chamber to account for non-specific binding of secondary antibodies. For screening of expression libraries we used the contrast method with either ArgC or PyrC as specificity control to determine the contrast via the formula:
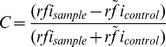
with 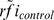 the median of all rfis of the control used.

### Sequencing

Clones harboring strong signals in microarray screening were selected to be sequenced. Sequencing was performed externally by LGC Genomics using HT7F (5′ ACATCGGCCCGGGTCTGAATC 3′) and FLXR (5′ CTTCCTTTCGGGCTTTGTTAG 3′) primers.

### Subcloning of positive candidates

After sequencing and identification of potentially immunodominant proteins, primers were designed to generate full-length clones for each identified gene, see Table S2 for a list of the primers used. Cloning was performed as mentioned above with slight modifications. The pFN18A vector was linearized using the following primer set; 18A IF linear for (5′ GTTTAAACGAATTCGGGCTC 3′) and 18A IF linear rev (5′ GGCGATCGCGTTATCGCTCTG 3′) with PCR conditions as mentioned before. Protein expression, lysis, and spotting of full-length proteins were performed as described above. The slides were incubated for 1 h at room temperature in a humidity chamber. For incubation with antibodies 3 Well or 16 Well ProPlate modules (Grace Biolabs) were attached to the HaloLink slides. Processing of the slides was done similar to the original screening, however several different antibodies were used, see section antibodies.

### ELISA

For testing of immunodominant characteristics with ELISA, the crude lysate was first purified using HaloLink magnetic beads (Promega) following the manufacturer's instructions. The proteins of interest were subsequently cleaved off by digestion with ProTEV protease (Promega) and concentration was determined by nanodrop measurements. The samples were diluted to a total protein content of 20 µg/ml in PBS and 50 µl of each sample was added to MaxiSorb Plates (Nunc). Each sample was analyzed at least in triplicate. The ELISA plate was covered with a lid and incubated overnight at 4°C in a humidity chamber. After five washing steps each with PBS+0.05% Tween-20 (PBST), the plates were blocked using 200 µl 5% non fat dried milk in PBS per well for 2 h. Afterwards, plates were washed three times with PBST. 100 µl of primary antibody solution (c = 4 µg/ml) in PBS containing 1% non fat dried milk were applied to each well using the respective desired antibody or PBS for controls. The plates were incubated for 2 h at room temperature and washed four times with PBST. Next, 100 µl of conjugated secondary antibody (Goat polyclonal to Rabbit IgG conjugated with Horseradish peroxidase, Abcam ab6721, c = 20 ng/ml) were added to each well and incubation carried on for 1 h. Finally, plates were washed once again four times with PBST and 100 µl 3,3′,5,5′-Tetramethylbenzidine (TMB, Sigma-Aldrich) was added to each well for detection. After 30 min of incubation at room temperature in the dark, the reaction was stopped by applying 100 µl of 2 M H_2_SO_4_ to each well. The optical density of each well was measured using the OMEGA Fluostar (BMG Labtech) at a wavelength of 450 nm.

### Bioinformatics

Primers were designed using Primer3 [37] within Geneious Pro 5.6.5 [38]. The sequenced inserts were identified by BLAST [39]. Peptide sequence secondary structures were predicted using the EMBOSS garnier [40] algorithm and the transmembrane regions predicted by TMHMM2.0 [30], [31]. Antigenic sites were predicted by EMBOSS antigenic [32], [33]. Data evaluation was performed by OriginPro 8 G (Originlab) and Microsoft Excel. 3-dimensional structure predictions were performed using the SWISS MODEL automated mode [41]–[45] and pdb files were visualized and analyzed by the UCSF Chimera package [46].Chimera is developed by the Resource for Biocomputing, Visualization, and Informatics at the University of California, San Francisco (supported by NIGMS P41-GM103311).

### Comparative data analysis

Analysis of full-length proteins was achieved by combining the results from ELISA and microarray data. Hence, the rfi of each sample was calculated. Next, a normalized rfi was generated by dividing the rfi of each sample by the median rfi of all the samples within an area of interest, i.e. incubation compartment. From these normalized rfis a median and standard deviation was calculated. If the median normalized rfi of the positive control was below the median normalized rfi of any of the negative references whilst taking the standard deviations into account, the test was rendered invalid. If the test passed the above criterion, the Q values were calculated as follows: 
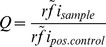
with 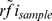 the median of normalized rfis of the sample and 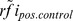 the median of the normalized rfis of the positive control OmpA respectively OmpF. The resulting error was calculated by error propagation according to Gauss. Finally, incorporating all valid tests, the mean Q value was determined along with its resulting error following error propagation by Gauss, see Table 1.

### Epitope Mapping

Several proteins were chosen for epitope mapping. These were the proteins encoded by KPN_00363, KPN_00459, KPN_00466, KPN_01100, KPN_01584, and KPN_02202. The proteins were divided into 15-mer oligopeptides with an overlap of 11 amino acids *in silico*. The synthesis and coupling to microarray slides was performed externally by JPT Peptide Technologies GmbH. Each peptide sequence was applied 9 times to one slide. The slides were used with Proplate 3-Well Chamber system (Grace) allowing for incubation with different antibodies. First, the slides were blocked with SuperBlock blocking buffer (Thermo Fischer) for 2 h, washed five times with PBS+0.05% Tween-20, primary antibodies applied, incubated overnight at 4°C with mild rocking, washed again, secondary antibodies applied for 2 h in the dark and after a final washing procedure, dried and scanned as above. Two different primary antibodies to K.*pneumoniae* were tested. The bottom chamber was always used as a control chamber, incubated only with secondary antibody.

### Alanine Scan

The peptide GAVVALSTTFA and 11 modifications thereof created by substituting one amino acid by alanine/glycine were synthesized by JPT Peptide Technologies GmbH. These peptides in combination with two peptides showing closely related sequences, GAVLALSSSFT and SAALALTSSFTE, were applied 9 times to slides. Incubation procedure was performed as described above for epitope mapping

### SDS-PAGE

The expression of the desired HaloTag fusion proteins was checked by SDS-PAGE. After lysis of cells, 2 µl of each protein extract was mixed with 1 µl of 10 µM HaloTag Alexa 488 ligand. After addition of 7 µL 1x TBS (100 mM Tris, 150 mM NaCl, pH 7.6) the reaction was incubated at room temperature for 30 minutes. 2 µl of each reaction were removed, mixed with 8 µl of 5x loading buffer (Fermentas) and 1 µl DTT and heated for 5 min at 70°C. The separation was performed on a Mini-Protean TGX Gel (Biorad, any kD, 15 wells) in a Protean II xi Cell chamber (Biorad) for 30 min at 200 V. As a size reference Benchmark Fluorescent Protein standard (Life Technologies) was used. Fluorescence was measured in a FLA-5100 (Fujifilm) with excitation at 473 nm.

## Supporting Information

Figure S1 SDS-PAGE of recombinantly expressed fusion constructs. Crude lysates labelled by incubation with HaloTag Alexa 488 ligand were separated by SDS-PAGE. M refers to the BenchMark Fluorescent Protein Standard (Invitrogen). The bands featuring the correct size are marked by red boxes. The proteins were as follows: 1 – KPN_02919, 2 – KPN_00466, 3 – KPN_02202, 4 – KPN_03356, 5 – KPN_00459, 6 – KPN_00182. The expression level is diverse, as KPN_00466 and KPN_00459 show weak bands, whereas KPN_02919 and KPN_02202 display bands of substantial intensity.
(TIF)

Figure S2 SDS-PAGE of recombinantly expressed fusion constructs II. Crude lysates labelled by incubation with HaloTag Alexa 488 ligand were separated by SDS-PAGE. M refers to the BenchMark Fluorescent Protein Standard (Invitrogen). The bands featuring the correct size are marked by red boxes. The proteins were as follows: 1 – KPN_00786, 2 – KPN_03732, 3-5 – KPN_02199, 6 – KPN_01100, 7 – KPN_03638, 8 – KPN_01784, 9 – KPN_00363, 10 – KPN_01584, 11 – hisJ, 12 – ompA, 13 – argC, 14 – gapA. Overall expression level is relatively weak, yet all bands are visible. KPN_01100 appears more intense than the candidate proteins.
(TIF)

Figure S3 Homology of GIAFGAVELFD of KPN_00459. The sequence derived from *K. pneumoniae* MGH78578 was used as a reference. The 100 best matches after BLAST analysis are shown in the figure with dots indicating identical residues. For differentiation of the sequences the NCBI accession number of the parent protein is given followed by the strain designation, if available. Only three *E. coli* strains in lines 63, 97 and 98 feature a valine residue at the second position instead of the consensus isoleucine. Consequently, this sequence is highly conserved within the Enterobacteriaceae including *E. coli, Klebsiella, Salmonella*, and *Enterobacter* among others.
(PDF)

Table S1 List of 192 sequenced clones after screening The clones were sequenced by LGC genomics using HT7F and FLX primers. Clones that were not successfully sequenced are indicated by “-“, clones carrying inserts too short to be reliably mapped to a gene are marked as truncated. Additionally, a few inserts were detected deriving from primer concatamers. These are displayed as “artificial”. The remaining clones are indicated by the corresponding locus tag and protein name. Several clones apparently carry identical inserts, especially obvious for KPN_01805 or KPN_02668. These were discarded from further analysis as the mapped inserts are very short and might have an artificial origin. Inserts that were highly unlikely to garner new immunogenic proteins, antigens described previously, e.g. OmpA, other molecules like tRNA and rRNA were abolished from further analysis.
(XLS)

Table S2 Primers used in this study. Each primer is given with a name, its sequence in 5′ to 3′ direction and the target gene or vector. For each target F represents forward and R the reverse primer. The primers were used for cloning in the In-Fusion SMARTer directional cDNA library construction kit.
(XLS)
