# Supplementary figures and images for: Identification of Antigenic Proteins of the Nosocomial Pathogen Klebsiella pneumoniae
Source: PLoS One. 2014 Oct 21;9(10):e110703. doi: 10.1371/journal.pone.0110703 (PMC4205017; doi:10.1371/journal.pone.0110703)

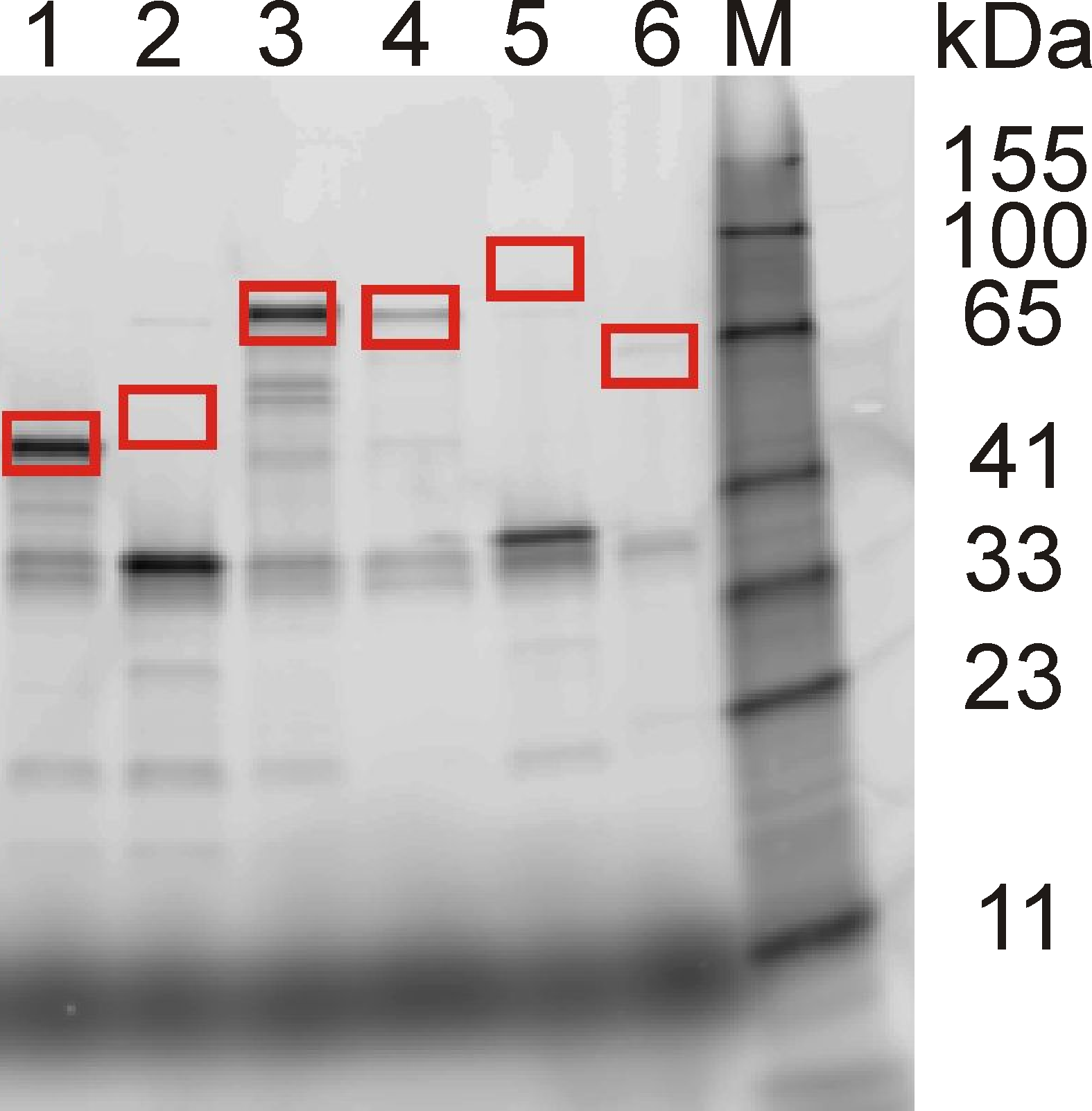

Supplement: Figure S1 — SDS-PAGE of recombinantly expressed fusion constructs. Crude lysates labelled by incubation with HaloTag Alexa 488 ligand were separated by SDS-PAGE. M refers to the BenchMark Fluorescent Protein Standard (Invitrogen). The bands featuring the correct size are marked by red boxes. The proteins were as follows: 1 – KPN_02919, 2 – KPN_00466, 3 – KPN_02202, 4 – KPN_03356, 5 – KPN_00459, 6 – KPN_00182. The expression level is diverse, as KPN_00466 and KPN_00459 show weak bands, whereas KPN_02919 and KPN_02202 display bands of substantial intensity. (TIF) [file pone.0110703.s001.tif]

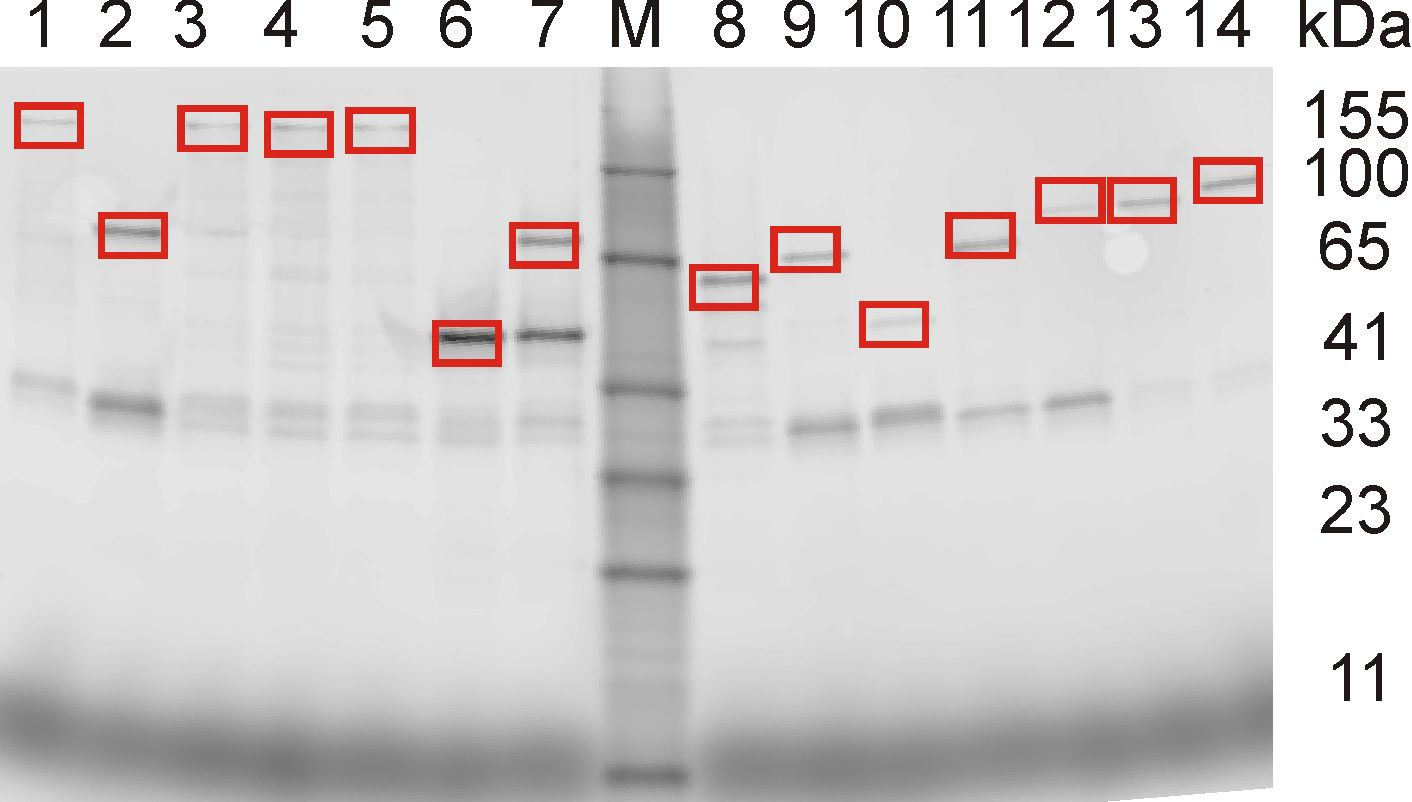

Supplement: Figure S2 — SDS-PAGE of recombinantly expressed fusion constructs II. Crude lysates labelled by incubation with HaloTag Alexa 488 ligand were separated by SDS-PAGE. M refers to the BenchMark Fluorescent Protein Standard (Invitrogen). The bands featuring the correct size are marked by red boxes. The proteins were as follows: 1 – KPN_00786, 2 – KPN_03732, 3-5 – KPN_02199, 6 – KPN_01100, 7 – KPN_03638, 8 – KPN_01784, 9 – KPN_00363, 10 – KPN_01584, 11 – hisJ, 12 – ompA, 13 – argC, 14 – gapA. Overall expression level is relatively weak, yet all bands are visible. KPN_01100 appears more intense than the candidate proteins. (TIF) [file pone.0110703.s002.tif]
