# Supplementary material for: Identification of Antigenic Proteins of the Nosocomial Pathogen Klebsiella pneumoniae
Source: PLoS One. 2014 Oct 21;9(10):e110703. doi: 10.1371/journal.pone.0110703 (PMC4205017; doi:10.1371/journal.pone.0110703)
Supplement: Figure S3 — Homology of GIAFGAVELFD of KPN_00459. The sequence derived from K. pneumoniae MGH78578 was used as a reference. The 100 best matches after BLAST analysis are shown in the figure with dots indicating identical residues. For differentiation of the sequences the NCBI accession number of the parent protein is given followed by the strain designation, if available. Only three E. coli strains in lines 63, 97 and 98 feature a valine residue at the second position instead of the consensus isoleucine. Consequently, this sequence is highly conserved within the Enterobacteriaceae including E. coli, Klebsiella, Salmonella, and Enterobacter among others. (PDF) [file pone.0110703.s003.pdf]

Identity

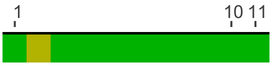

Sequence	Alignment
1. YP_001334140 (Klebsiella pneumoniae subsp. pneumoniae MGH 78578)	G I A F G A V E L F D
2. ZP_16338573 (Klebsiella pneumoniae subsp. pneumoniae ST258-K26BO)	. . . .
3. WP_000546198 (Salmonella enterica)	. . . .
4. WP_000546210 (Salmonella enterica)	. . . .
5. YP_151428 (Salmonella enterica subsp. enterica serovar Paratyphi A str. ATCC 9150)	. . . .
6. WP_000546200 (Salmonella enterica)	. . . .
7. NP_806099 (Salmonella enterica subsp. enterica serovar Typhi str. Ty2)	. . . .
8. WP_000546211 (Salmonella enterica)	. . . .
9. WP_000546206 (Salmonella enterica)	. . . .
10. WP_000546204 (Salmonella enterica)	. . . .
11. WP_000546257 (Escherichia coli)	. . . .
12. WP_001523355 (Salmonella enterica)	. . . .
13. WP_000546217 (Salmonella enterica)	. . . .
14. ZP_05968945 (Enterobacter cancerogenus ATCC 35316)	. . . .
15. YP_004592798 (Enterobacter aerogenes KCTC 2190)	. . . .
16. ZP_16315499 (Salmonella enterica subsp. enterica serovar Senftenberg str. SS209)	. . . .
17. NP_459487 (Salmonella enterica subsp. enterica serovar Typhimurium str. LT2)	. . . .
18. YP_003227592 (Escherichia coli O26:H11 str. 11368)	. . . .
19. WP_000546205 (Salmonella enterica)	. . . .
20. YP_668467 (Escherichia coli 536)	. . . .
21. YP_215521 (Salmonella enterica subsp. enterica serovar Choleraesuis str. SC-B67)	. . . .
22. NP_455088 (Salmonella enterica subsp. enterica serovar Typhi str. CT18)	. . . .
23. WP_000546258 (Escherichia albertii)	. . . .
24. YP_002225600 (Salmonella enterica subsp. enterica serovar Gallinarum str. 287/91)	. . . .
25. YP_005018576 (Klebsiella oxytoca KCTC 1686)	. . . .
26. ZP_12380657 (Shigella flexneri 2747-71)	. . . .
27. WP_001657858 (Escherichia coli)	. . . .
28. ZP_10806792 (Enterobacter sp. SST3)	. . . .
29. WP_001494008 (Escherichia coli)	. . . .
30. WP_000546208 (Salmonella enterica)	. . . .
31. ZP_23927799 (Klebsiella pneumoniae 700603)	. . . .
32. YP_004729343 (Salmonella bongori NCTC 12419)	. . . .
33. WP_001499490 (Escherichia coli)	. . . .
34. WP_001460559 (Escherichia coli)	. . . .
35. NP_752532 (Escherichia coli CFT073)	. . . .
36. ZP_16276836 (Citrobacter freundii ATCC 8090 = MTCC 1658)	. . . .
37. WP_000546247 (Escherichia coli)	. . . .
38. WP_001582195 (Escherichia coli)	. . . .
39. WP_000546227 (Escherichia coli)	. . . .
40. YP_006476514 (Enterobacter cloacae subsp. dissolvens SDM)	. . . .
41. YP_007845910 (Enterobacter cloacae subsp. cloacae NCTC 9394)	. . . .
42. WP_001688478 (Escherichia coli)	. . . .
43. ZP_04560942 (Citrobacter sp. 30_2)	. . . .
44. YP_004827522 (Enterobacter asburiae LF7a)	. . . .
45. ZP_06353937 (Citrobacter youngae ATCC 29220)	. . . .
46. ZP_19695435 (Escherichia coli KTE193)	. . . .
47. YP_003611758 (Enterobacter cloacae subsp. cloacae ATCC 13047)	. . . .
48. WP_001535126 (Escherichia coli)	. . . .
49. ZP_14870777 (Shigella flexneri 1235-66)	. . . .
50. WP_000546194 (Escherichia sp. TW09231)	. . . .
51. NP_286219 (Escherichia coli O157:H7 str. EDL933)	. . . .
52. WP_001695449 (Escherichia coli)	. . . .
53. YP_002240021 (Klebsiella pneumoniae 342)	. . . .
54. YP_002327989 (Escherichia coli O127:H6 str. E2348/69)	. . . .
55. ZP_06014806 (Klebsiella pneumoniae subsp. rhinoscleromatis ATCC 13884)	. . . .
56. YP_002381718 (Escherichia fergusonii ATCC 35469)	. . . .
57. ZP_08497148 (Enterobacter hormaechei ATCC 49162)	. . . .
58. WP_000546249 (Escherichia coli)	. . . .
59. WP_001674073 (Escherichia coli)	. . . .
60. YP_001571445 (Salmonella enterica subsp. arizonae serovar 62:z4,z23:- str. RSK2980)	. . . .
61. YP_001454214 (Citrobacter koseri ATCC BAA-895)	. . . .
62. YP_006577589 (Enterobacter cloacae subsp. cloacae ENHKU01)	. . . .
63. WP_001529771 (Escherichia coli)	. V . . . .
64. WP_001732629 (Escherichia coli)	. . . .
65. YP_004950875 (Enterobacter cloacae EcWSU1)	. . . .
66. YP_002396553 (Escherichia coli ED1a)	. . . .
67. ZP_17106706 (Klebsiella oxytoca 10-5246)	. . . .
68. WP_000546233 (Escherichia coli)	. . . .
69. ZP_09036574 (Enterobacter mori LMG 25706)	. . . .
70. WP_001634070 (Escherichia coli)	. . . .
71. WP_001730470 (Escherichia coli)	. . . .
72. ZP_10891501 (Klebsiella sp. OBRC7)	. . . .
73. ZP_12355085 (Shigella flexneri VA-6)	. . . .
74. WP_001506953 (Escherichia coli)	. . . .
75. YP_001879187 (Shigella boydii CDC 3083-94)	. . . .
76. YP_309476 (Shigella sonnei Ss046)	. . . .
77. ZP_03064974 (Shigella dysenteriae 1012)	. . . .
78. WP_000546243 (Escherichia coli)	. . . .
79. YP_003220456 (Escherichia coli O103:H2 str. 12009)	. . . .
80. WP_000546231 (Escherichia coli)	. . . .
81. WP_001503913 (Escherichia coli)	. . . .
82. WP_001501086 (Escherichia coli)	. . . .
83. ZP_17100634 (Klebsiella oxytoca 10-5245)	. . . .
84. WP_001667400 (Escherichia coli)	. . . .
85. WP_000546242 (Escherichia coli)	. . . .
86. NP_706371 (Shigella flexneri 2a str. 301)	. . . .
87. WP_001616532 (Escherichia coli)	. . . .
88. YP_002411280 (Escherichia coli UMN026)	. . . .
89. YP_003364170 (Citrobacter rodentium ICC168)	. . . .
90. ZP_02902286 (Escherichia albertii TW07627)	. . . .
91. ZP_17115517 (Klebsiella oxytoca 10-5250)	. . . .
92. WP_000546250 (Escherichia coli)	. . . .
93. YP_002291778 (Escherichia coli SE11)	. . . .
94. WP_001497395 (Escherichia coli)	. . . .
95. ZP_10490673 (Enterobacter radicincitans DSM 16656)	. . . .
96. WP_001714934 (Escherichia coli)	. . . .
97. WP_000546239 (Escherichia coli)	. V . . . .
98. NP_415011 (Escherichia coli str. K-12 substr. MG1655)	. V . . . .
99. ZP_17094756 (Klebsiella oxytoca 10-5243)	. . . .
100. WP_000546195 (Escherichia albertii)	. . . .
